# Therapeutic effectiveness of iodine-rich herbs in treating Graves’ hyperthyroidism: a retrospective cohort study from a single center

**DOI:** 10.3389/fendo.2025.1573617

**Published:** 2025-08-11

**Authors:** Yiwen Lai, Mengfei Yang, Jing Li, Di Gan, Qingyang Liu, Yingna Wang, Tianshu Gao

**Affiliations:** ^1^ Department of Endocrinology, The Affiliated Hospital of Liaoning University of Traditional Chinese Medicine, Shenyang, Liaoning, China; ^2^ Graduate School, Liaoning University of Traditional Chinese Medicine, Shenyang, Liaoning, China; ^3^ Department of Endocrinology, The First Hospital of China Medical University, Shenyang, Liaoning, China

**Keywords:** iodine-rich herbs, Grave’s hyperthyroidism, China, retrospective, cohort

## Abstract

**Objective:**

To evaluate the effectiveness of the traditional Chinese medicine decoction with iodine-rich herbs as the main agent (IRH) in patients with Graves’ hyperthyroidism (GD).

**Methods:**

A retrospective cohort study was conducted in a tertiary A traditional Chinese medicine hospital in northeastern China. We followed the effectiveness and safety of IRH in treating GD patients from January 2010 to August 2024 through the Intelligent Research Data Platform. Patients treated with IRH were classified into the IRH cohort, and those treated with antithyroid drugs (ATD) were classified into the ATD cohort. The characteristics of the two groups were balanced using propensity score matching (PSM). We used logistic regression, the Kaplan-Meier method, and the Cox proportional hazard model to compare the efficacy differences between IRH and ATD and to preliminarily identify the influencing factors of IRH efficacy through subgroup analysis of clinical characteristics.

**Results:**

After screening and PSM, 73 GD patients treated with IRH and 73 with ATD were included in this study. IRH could significantly improve serum free T3 (fT3), free T4 (fT4), thyroid-stimulating hormone (TSH), and TSH receptor antibody (TRAb) levels in GD patients. Among the 73 patients who received IRH, the serum fT3, fT4, and TSH levels of 63 (86.3%), 65 (89%), and 40 (54.8%) patients, respectively, returned to normal, and the efficacy of IRH persisted for 50 (68.5%) patients. In addition, the median time to normalization and the regression analysis after correction for confounding factors did not show significant differences between IRH and ATD regarding efficacy and persistence. The safety evaluation results of the two drugs were similar. Higher IRH doses (>= 40g) may improve efficacy, while younger age, male gender, goiter, and more severe thyrotoxicosis might lead to poor efficacy of IRH.

**Conclusion:**

Compared with ATD, IRH could also significantly improve the serum fT3, fT4, TSH, and TRAb levels of patients with GD and has a comparable duration of efficacy. For patients with mild to moderate GD, IRH provided a safe as well as effective alternative therapy.

## 1 Introduction

Thyrotropin receptor antibodies arise in Graves’ hyperthyroidism (GD), an organ-specific autoimmune disorder that triggers an overproduction of thyroid hormone synthesis and release. The prevalence of GD has steadily declined in China following 20 years of universal salt iodization because the iodine nutritional status of Chinese people has been improved, and the recent epidemiological survey reported that the prevalence was 0.53% (1). However, this implies that about seven million people still have GD. In China, many patients are hesitant to undergo lifelong LT4 replacement therapy for permanent hypothyroidism and refuse surgery or radioiodine. Additionally, the side effects of antithyroid drugs (ATD), which can include granulocytopenia, ANCA vasculitis, and liver damage, have further decreased patients’ willingness to pursue these treatments (2). As a result, there is a growing expectation for additional treatment strategies, in which traditional Chinese medicine therapies are being considered.

Herbal medicine is the main form of treatment in traditional Chinese medicine. Haizao (Sargassum) and Kunbu (Laminaria japonica), according to *Shennong’s Classic of the Materia Medica* (completed around the 1st century BC) and *Mingyi Bielu* (completed around 2000 years ago), can “master Ying diseases, the nucleus of the neck, and tumor qi.” Haizao and Kunbu are commonly referred to as “iodine-rich herbs” in reference to their high iodine content (3, 4). The first record of IRH treatment of “Ying diseases” was in *Zhou Hou Bei Ji Fang*. “Ying diseases” is a general term for thyroid diseases characterized by goiter, so the treatment of IRH has been extended to GD and thyroid cancer (5–7). In addition to suppressing autoimmune antibody levels in rats with thyroid disease (8–10), prior pharmacological studies have discovered that Haizao can prevent apoptosis and cell proliferation (11). Kunbu incorporates several therapeutic advantages, which include anti-inflammatory, antioxidant, apoptosis-blocking, angiogenesis-inhibition, and anti-cell proliferation qualities (12, 13). The network pharmacology results of IRH and its common traditional Chinese medicine decoction for treating hyperthyroidism show that their intervention mechanism may be regulating cell proliferation, regulating kinase activity, restricting pro-inflammatory cytokines, and restoring equilibrium immunity (14, 15).

Some previous single-center RCTs and meta-analyses have proved that Chinese herbal decoctions containing IRH are effective for treating GD (7, 16–19). Consequently, the guidelines (2023) and consensus (2021) recommend employing IRH to treat GD (20, 21). Additionally, prior multicenter, randomized, double-blind, placebo-controlled clinical trials conducted by the research team have determined that IRH-containing traditional Chinese medicine decoction could enhance the life quality for GD patients by substantially decreasing serum free T3 (fT3), free T4 (fT4), TSH receptor antibody (TRAb), and superior thyroid artery peak systolic velocity (22). However, the guidelines recommend a dosage range for IRH, and dosage adjustment of drugs is common in clinical practice. Retrospective cohort studies on the clinical application of IRH in real-world settings are still lacking.

Therefore, to refine the clinical evidence of IRH-based herbal decoction intervention for GD, we retrospectively conducted the effectiveness and safety of selected GD patients who applied IRH using a single-center hospital data platform.

## 2 Methods

### 2.1 Study design

To evaluate the effectiveness of IRH in the treatment of GD, we performed a retrospective cohort study at a comprehensive tertiary A Chinese medicine hospital. This trial protocol was approved by the Ethics Committee of the Affiliated Hospital of Liaoning University of Traditional Chinese Medicine (Y2024199CS(KT)-199-01) and registered at International Traditional Medicine Clinical Trial Registry (registration number: ITMCTR2025000216) following the *Declaration of Helsinki*, the *Good Clinical Practice*, and relevant regulations.

### 2.2 Participants

Eligible subjects were GD patients who had received IRH or ATD and presented to the Department of Endocrinology, Affiliated Hospital of Liaoning University of Traditional Chinese Medicine from January 2010 to August 2024. Patients with positive TRAb, decreased serum thyroid-stimulating hormone (TSH) levels, along with elevated serum fT4 and/or fT3 levels, have been identified with GD (23). Patients who had not received IRH, or cross-medication, were younger than 18 years old, were pregnant or planning to become pregnant, had a thyrotoxic crisis, chronic alcohol abuse, drug dependence, or a mental illness, were not included in the study. In a real-world setting, IRH and ATD can be performed in combination or crossed over for the treatment of GD. However, in order to compare the effect of IRH alone with ATD, the group of co-treated patients was also excluded from this study. Patient information was collected through the hospital’s intelligent research data platform V1.0.0 (Hangzhou Yitu Medical Technology Co., Ltd.), and patients treated with IRH were classified into the IRH group and those treated with ATD were classified into the ATD group based on whether IRH treatment was used as an exposure factor. This study used unidentifiable data for research purposes, and the subject could not be located. In addition, informed consent and informed consent signatures were waived since the project excluded economic interests or personal privacy.

Since neither the IRH nor the ATD groups were matched with the patients randomly, the distribution of covariates in the two groups was balanced using propensity score matching (PSM) to reduce bias. We used a multivariable logistic regression model to calculate the propensity score for each individual. The model included the following baseline attributes as covariates: age, gender, fT3 level, fT4 level, TSH level, and TRAb level. Based on the propensity score, the 1:1 nearest neighbor matching method was used using the MatchIt package in RStudio, and the standardized mean difference (SMD) < 0.1 and *t* test were used to verify the group balance. The matched data was used to estimate the effect of IRH on intervention. We collected patients from the hospital’s data platform and performed PSM. Since the reliability of the research results had to be ensured, the data of a single center were analyzed retrospectively to include as many patients as possible who met the requirements, so a sample size calculation could not be performed in advance.

### 2.3 Study interventions

IRH includes Haizao and/or Kunbu, processed Chinese herbal pieces decocted in water when used. ATD is either methimazole (MMI) or propylthiouracil (PTU) and is available in tablet form. The data platform records the start time, dosage, number of days, and frequency of administration for each patient.

### 2.4 Outcomes and variables

We assessed the efficacy of IRH by measuring serum fT3, fT4, TSH, and TRAb levels after treatment, recording the normalization of fT3, fT4, and TSH and the time required for normalization, and evaluating the persistence of efficacy by whether the post-treatment fT3 and/or fT4 level was higher than the baseline (24). The event start time was the date of the previous measurement before the first intervention. The end time was recorded as the date of the last laboratory test with a record, the date of normalization of serum fT3, fT4, or TSH, and the date of the first laboratory test in which the serum fT3 and/or fT4 levels first exceeded the baseline level, respectively. Blood test results residing within the normal range were referred to as normalization.

The main potential GD-related confounders included age, gender, goiter, serum fT3, fT4, and TRAb level measured at baseline (25). We evaluated the presence of goiter based on the thyroid ultrasound results (26). We also recorded the baseline anti-thyroid peroxidase antibody (TPOAb) and anti-thyroglobulin antibody (TgAb) levels, initial IRH dose, and duration of IRH administration.

Since liver function damage is a common adverse event of ATD, this study evaluated the safety of IRH based on alanine transaminase (ALT), aspartate transaminase (AST), γ-glutamyl transpeptidase (GGT), alkaline phosphatase (ALP), and total bilirubin (TBIL) levels to estimate its safety (22).

### 2.5 Data measurement

Serum fT3, fT4, TSH, TRAb, TPOAb, and TgAb levels were measured using electrochemiluminescence immunoassay (ECLusys, Roche Diagnostics GmbH, Mannheim, Germany). The reference values in this study were as follows: serum fT4, 12–22 pmol/L; fT3, 3.1-6.8 pmol/L; TSH, 0.27-4.2 uIU/ml; TRAb, ≤ 1.75 IU/L; TPOAb, ≤ 34 IU/ml; TgAb, ≤ 115 IU/ml. The automatic biochemical analyzer with spectrophotometry (Hitachi High-Tech, Tokyo, Japan) was utilized to evaluate liver function. Meanwhile, a thyroid color Doppler ultrasound (12–18 MHz linear converter) was employed.

### 2.6 Statistical analysis

The χ^2^ test, continuously corrected χ^2^ test, or Pearson’s χ^2^ test was employed to compare proportions. Non-normally distributed quantitative data were expressed as the median and range. The Mann-Whitney U test or the Wilcoxon matched-pairs signed rank test was utilized to compare the groups.

To further compare the effects of the two drugs on the normalization of fT3, fT4, or TSH and the persistence of the efficacy in GD patients, we used a multivariate logistic regression analysis to obtain the adjusted odd ratio (aOR) and 95% CI, performed a Log-rank test, plotted a Kaplan-Meier curve, and used a Cox proportional hazard regression model to calculate the adjusted hazard ratio (aHR) and 95% CI. If the proportional hazards assumption is invalid, Cox regression with time-dependent covariates is performed using the timecox function of the R timereg package. Applying the resampling technique, the timecox function allows for a Cox model with time-fixed and time-varying coefficients (27, 28).

We performed 3 additional sensitivity analyses. First, different covariates were corrected to confirm whether other potential confounding factors affect the regression results, and the unadjusted (crude) results were also reported. Second, to study the intervention effect of IRH on thyroid function, we regarded the combined normalcy of fT3 and fT4 as a composite outcome and analyzed it similarly. Finally, a multiple-factor regression analysis was carried out directly on the enrolled population without PSM to explore the results of different statistical model corrections (29).

Data collection and collation were applied using Excel, and all data processing and graphing were performed using Graphpad Prism 8, SPSS 26.0, or RStudio 2024.04.2. A two-tailed *p*-value of less than 0.05 is considered to be statistically significant.

## 3 Results

### 3.1 Patient selection process and basic demographic information

**Figure 1:**
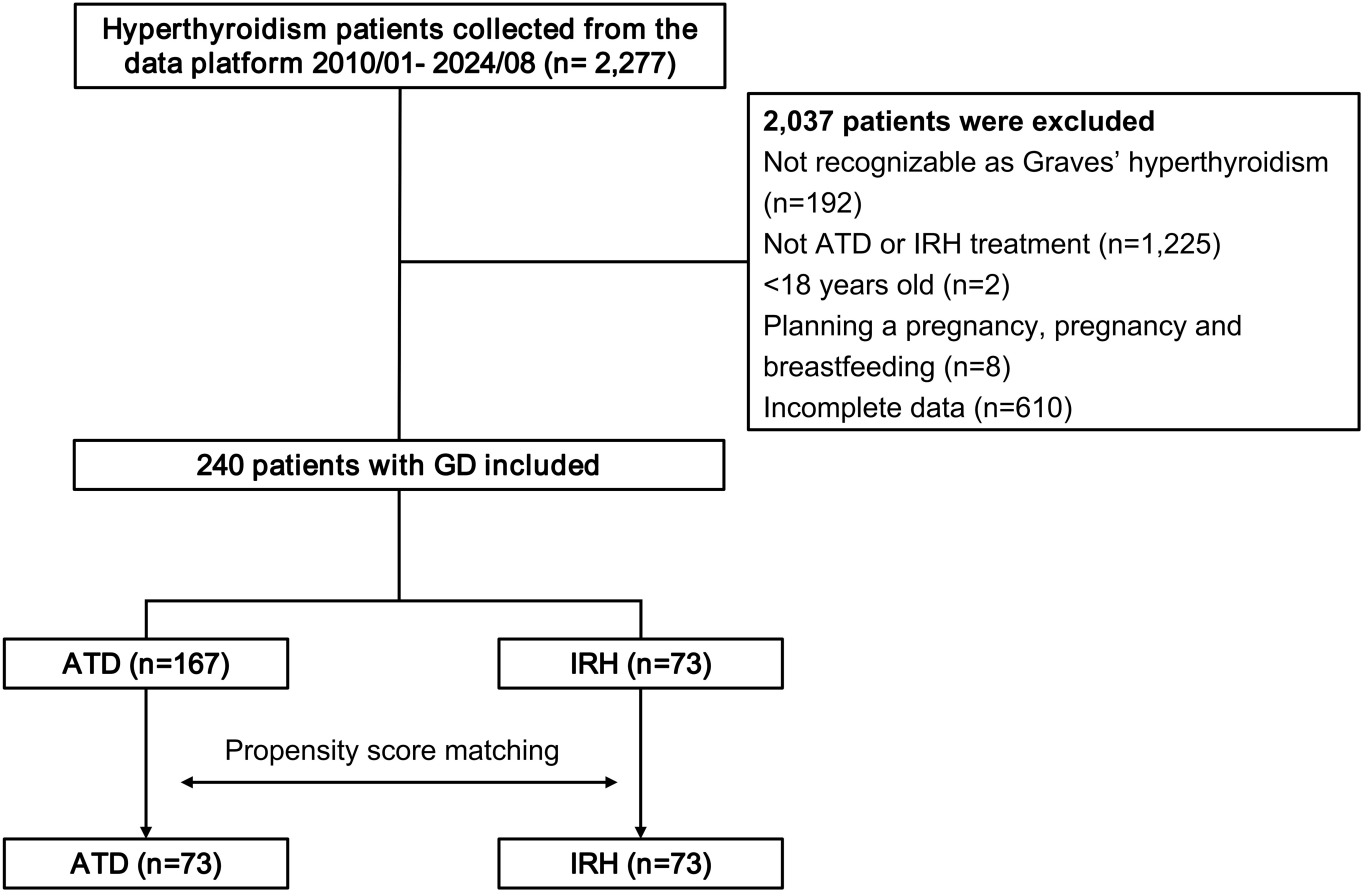
Flowchart of the study population.

This study retrospectively collected 2,277 patients with a definite diagnosis of GD from the evidence-based medical research platform. Following removing patients who did not fit the requirements, 240 eligible cases were treated with IRH or ATD alone (**Figure 1**).

**Figure 2:**
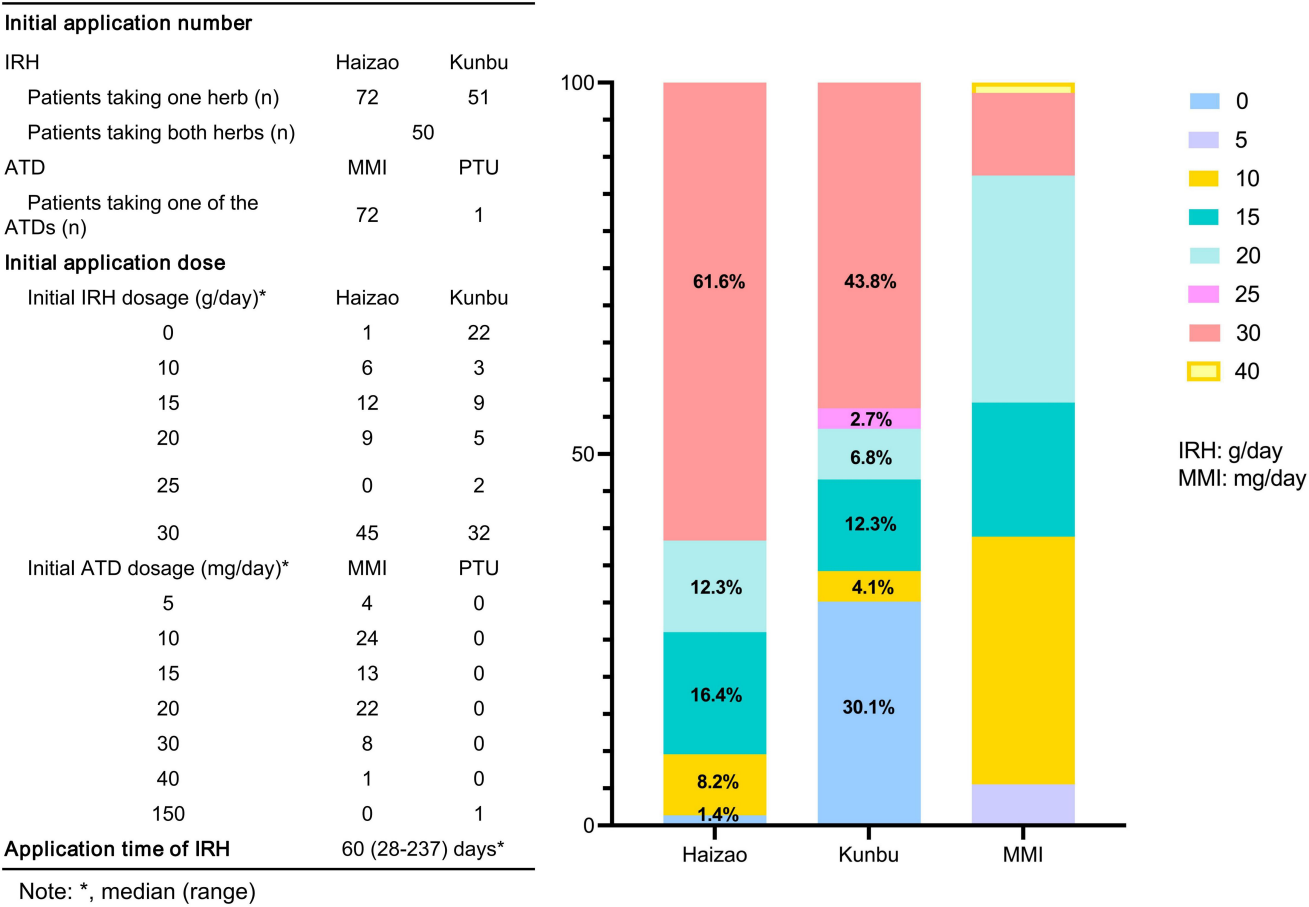
Description of the initial dosing scheme for the two groups of patients. MMI, methimazole; PTU, propylthiouracil.

Compared with patients in the ATD group, the IRH group had milder thyrotoxicosis and antibody levels. We implemented PSM to create 73 IRH-treated and 73 ATD-treated patients to eradicate the selection or propensity biases statistically. **Table 1** described the baseline demographic features of the two treatment groups, and the baseline characteristics after matching were comparable. **Figure 2** showed the distribution of initial doses in the cohort studied. The table on the left describes the number of people starting different dosing regimens and the median doses. Of the 73 patients using ATD, one applied PTU tablets and 72 applied MMI tablets.

**Table 1:** Baseline characteristics of patients with GD treated with ATD or IRH.

Clinical Data	Pre-match				Post-PSM				
	Total (n=240)	ATD (n=167)	IRH (n=73)	*P*	Total (n=146)	ATD (n=73)	IRH (n=73)	*P*	SMD
Gender (male/female)	48/192	37/130	11/62	.207	26/120	15/58	11/62	.516	-0.1532
Age (years)	39 (18-84)	39 (11-84)	42 (21-70)	.21	41 (18-80)	40 (18-80)	42 (21-70)	.967	-0.0448
Goiter (yes/no)	81/131	60/107	21/24	.188	43/75	22/51	21/24	.106	
fT3 (pmol/L)	16.31 (4.73-56.57)	18.15 (5.63-56.57)	12.2 (4.73-47.17)	.001	12.19 (4.73-50)	11.94 (5.63-50)	12.2 (4.73-47.17)	.998	0.0222
fT4 (pmol/L)	41.45 (13.42-128.7)	49.25 (13.42-128.7)	31.13 (13.91-100)	<.001	32.68 (13.42-100)	33.69 (13.42-100)	31.13 (13.91-100)	.479	-0.0366
TSH (uIU/ml)	0.0049 (-0.006-0.138)	0.0049 (-0.006-0.138)	0.0049 (-0.006-0.124)	.539	0.0049 (-0.006-0.138)	0.0049 (-0.006-0.138)	0.0049 (-0.006-0.124)	.831	0.1621
TRAb (IU/L)	9.66 (1.08-300)	10.36 (1.89-300)	7.87 (1.08-74.02)	.042	8.48 (1.91-40.01)	8.48 (1.91-40.01)	7.87 (1.08-74.02)	.86	-0.0111
TPOAb (positive/negative)	113/51	85/33	28/18	.165	63/33	35/15	28/18	.468	
TgAb (positive/negative)	92/73	67/52	25/21	.821	50/46	25/25	25/21	.825	
Total consultation time (days)	224.5 (1-2450)	258 (1-2450)	170 (6-1497)	.006	200.5 (6-1497)	216 (9-1419)	170 (6-1497)	.092	

At the initial application of IRH treatment, 72 out of 73 cases took Haizao, 51 took Kunbu, and 50 chose both. The bar chart on the right showed the proportion of the initial dose of Haizao, Kunbu, and MMI. 30 g of Haizao and Kunbu accounted for most of the initial dose (Haizao: 61.6%, Kunbu: 43.8%).

### 3.2 IRH significantly improved the serum fT3, fT4, TSH, and TRAb levels in GD patients

**Figure 3:**
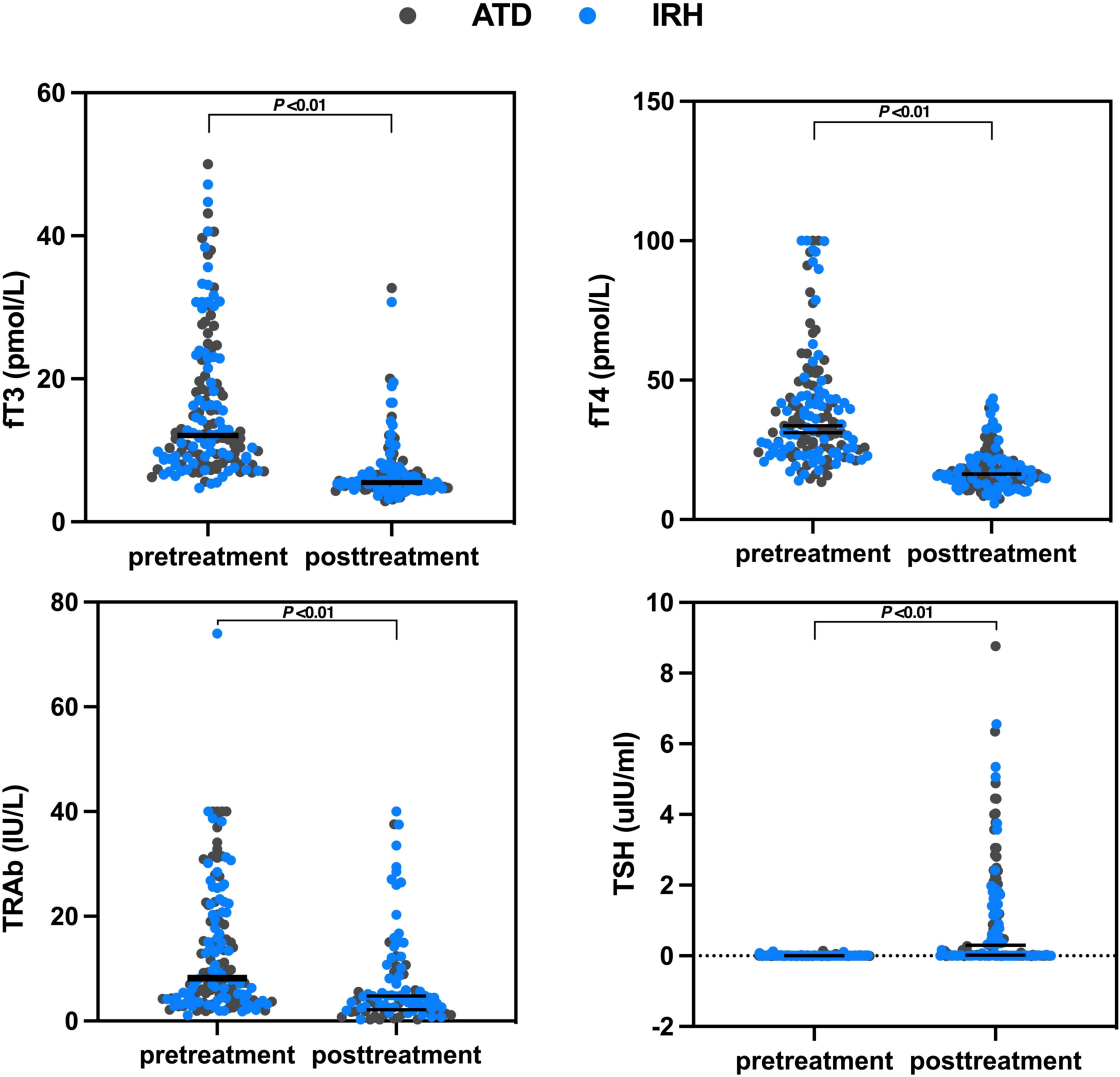
Changes in serum fT3, fT4, TSH, and TRAb levels before and after treatment in the two groups of patients.

We collected serum fT3, fT4, TSH, and TRAb levels before treatment and at the time of the last medical record. The median days of application were 100 days (range: 33–778 days) and 60 days (range: 28–237 days) for ATD and IRH, respectively. After comparing the results, we observed that the intervention effect of IRH on GD patients is homogeneous to ATD (**Table 2**, **Figure 3**).

**Table 2:** Changes in serum fT3, fT4, TSH, and TRAb levels before and after treatment in the two groups of patients.

Treatment	Peri-treatment	fT3 (pmol/L)		fT4 (pmol/L)		TSH (uIU/ml)		TRAb (IU/L)	
		median	*P*	median	*P*	median	*P*	median	*P*
ATD	Pretreatment	11.94 (5.63-50)	<.001	33.69 (13.42-100)	<.001	0.0049 (0-0.138)	<.001	8.48 (1.91-40.01)	<.001
	Posttreatment	5.39 (2.9-32.7)		16.4 (7.45-40.07)		0.299 (0-8.76)		2.14 (0.29-37.57)	
IRH	Pretreatment	12.2 (4.73-47.17)	<.001	31.13 (13.91-100)	<.001	0.0049 (0-0.124)	<.001	7.87 (1.08-74.02)	.005
	Posttreatment	5.6 (3.2-30.72)		16.23 (5.74-43.49)		0.041 (0-6.56)		4.76 (0.29-40)	

### 3.3 The response of GD patients to IRH or ATD treatment

#### 3.3.1 The effect of IRH on the normalization of serum fT3, fT4, and TSH in GD patients was comparable to ATD

**Figure 4:**
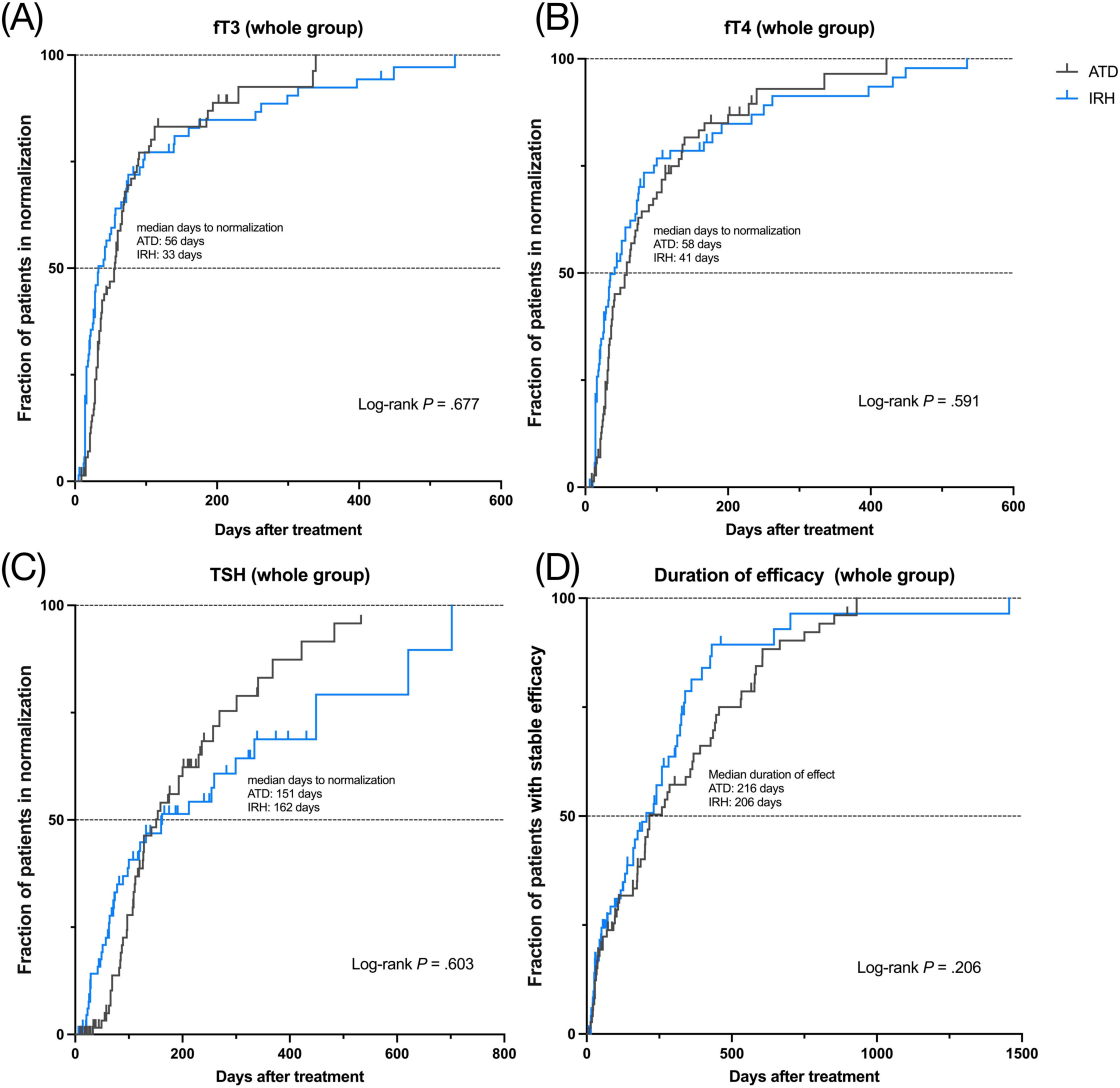
Kaplan-Meier curves of two drugs for 146 GD patients, median normalization time or median duration, and Log-rank test. **(A)**. fT3 normalization; **(B)**. fT4 normalization; **(C)**. TSH normalization; **(D)**. persistence of drug efficacy.

To better compare the efficacy of IRH and ATD, we marked patients whose serum fT3, fT4, or TSH had returned to normal. There was no discernible difference in the distribution of patients between the IRH and the ATD group (fT3: *p* = 1, fT4: *p* = .461, TSH: *p* = .503). Likewise, the logistic regression model after multiple covariate corrections also suggested the similarity of the efficacy of the two drugs (**Table 3**). We further calculated the time required for all patients to achieve normal serum fT3, fT4, or TSH, plotted Kaplan-Meier curves, performed Log-rank tests, and constructed a multivariate Cox regression model (**Figures 4A-C**, **Table 3**). The results still advised that the effect of IRH on the normalization of fT3, fT4, and TSH resembled ATD.

**Table 3:** Efficacy and persistence of IRH and ATD interventions in GD patients.

Group	Participants, No.	Events, No. (%)	χ^2^ *P*	logistic regression								Cox regression	
				Crude		model 1		model 2		model 3		*P*	aHR (95% CI)
				*P*	OR (95%CI)	*P*	aOR (95%CI)	*P*	aOR (95%CI)	*P*	aOR (95%CI)		
fT3													
ATD	63	73 (86.3)	1	Reference									
IRH	63	73 (86.3)		1	1 (0.389-2.569)	.636	1.325 (0.413-4.249)	.999	1 (0.387-2.582)	.622	1.348 (0.411-4.421)	.452	1.201 (0.803-1.795)
fT4													
ATD	62	73 (84.9)	0.461	Reference									
IRH	65	73 (89.0)		.462	1.442 (0.544-3.821)	.212	2.426 (0.603-9.764)	.482	1.424 (0.532-3.81)	.207	2.535 (0.597-10.762)	.627	1.12 (0.742-1.69)
TSH													
ATD	44	73 (60.3)	0.503	Reference									
IRH	40	73 (54.8)		.503	0.799 (0.414-1.542)	.467	0.753 (0.35-1.619)	.537	0.81 (0.415-1.582)	.553	0.791 (0.364-1.719)	.182	0.722 (0.422-1.235)
persistence of efficacy													
ATD	59	73 (80.8)	0.128	Reference									
IRH	50	73 (68.5)		.089	0.516 (0.24-1.107)	.12	0.48 (0.191-1.21)	.115	0.518 (0.229-1.173)	.176	0.504 (0.186-1.361)	.993	1.002 (0.629-1.594)

Crude, the model only used drug intervention as the independent variable, i.e., unadjusted for confounding factors. In model 1, covariates included age, gender, and goiter; in model 2, covariates were initial fT3, initial fT4, and initial TRAb levels; in model 3, covariates were the integration of model 1 and model 2. The covariates corrected in the multivariate Cox regression model included age, gender, goiter, initial fT3, initial fT4, and initial TRAb levels.

#### 3.3.2 The sustainability of IRH efficacy was consistent with ATD

If the serum fT3 and/or fT4 level after treatment was higher than the baseline, it was considered to have poor persistence of drug efficacy and vice versa. We recorded the patients with good persistence and duration in 146 patients and analyzed them using the same method. **Table 3** and **Figure 4D** indicated that no significant difference exists between the persistence of IRH and ATD in GD patients in this study.

### 3.4 Adverse events

We separately recorded the proportion of patients with abnormalities in ALT, AST, ALP, GGT, and TBIL after administration. Adverse events were not considered to have occurred when medical records showed that the patient’s safety markers were transiently abnormal during treatment and eventually returned to normal without discontinuation. This study found less GGT impairment with IRH than ATD (*p*=.007), and only slightly more AST abnormalities than with ATD (ATD vs IRH: 1% vs 4%), suggesting that IRH was safer for GD patients (**Table 4**).

**Table 4:** Summary of reported adverse events.

Adverse events	ATD (n=73)	IRH (n=73)	*P*
ALT abnormalization	7/73 (10%)	4/73 (5%)	.531
AST abnormalization	1/73 (1%)	3/73 (4%)	.612
GGT abnormalization	11/73 (15%)	1/73 (1%)	.007
ALP abnormalization	5/73 (7%)	1/73 (1%)	.211
TBIL abnormalization	0/73 (0%)	1/73 (1%)	1

### 3.5 Predictive factors for the efficacy of IRH

Although our results have demonstrated that IRH has efficacy and safety indistinguishable from ATD, exploring the factors affecting its effectiveness is still considerable. We preliminarily explored the variables influencing the potency of IRH through subgroup analysis to confirm the improvement of fT3, fT4, TSH, and TRAb levels after IRH intervention in different populations. **Table 5** displayed that IRH could significantly improve the serum fT3, fT4, and TSH levels in different population subgroups, indicating that the improvement of thyrotoxicosis by IRH was stable. The effect of IRH in reducing TRAb levels might vary from person to person. When the patient was younger than 41 years old, had a goiter, and had higher fT3 and fT4 levels, the improvement of TRAb levels by IRH was not significant. We observed a substantial intervention effect of IRH in patients with GD with baseline TRAb levels >= 8.48 IU/L (*p* = .008). In addition, we found that patients with GD who received a larger initial IRH dose and a longer IRH application time could achieve more effective TRAb improvement.

**Table 5:** Improvement of serum fT3, fT4, TSH, and TRAb after IRH application in different subgroups of the population.

IRH group	Peri-treatment	fT3 (pmol/L)		fT4 (pmol/L)		TSH (uIU/ml)		TRAb (IU/L)	
Age (median)									
< 41years	Pretreatment	11.4 (5.34-35.6)	<.001	29.9 (13.9-99.9)	<.001	0.0049 (0-0.12)	<.001	6.54 (1.08-26.8)	.635
	Posttreatment	5.98 (3.66-30.7)		17.7 (10.5-42.1)		0.01 (0-5.35)		4.91 (0.8-37.5)	
≥ 41years	Pretreatment	14 (4.73-47.2)	<.001	33.1 (17.3-100)	<.001	0.0049 (0-0.11)	<.001	9.59 (2.08-74)	.001
	Posttreatment	5.33 (3.2-19)		14.9 (5.74-43.5)		0.37 (0-6.56)		4.44 (0.29-40)	
Gender									
male	Pretreatment	11 (7.01-35.6)	.032	28.9 (13.9-51)	.032	0.0049 (0-0.01)	.031	20.3 (3.11-26.8)	.578
	Posttreatment	5.53 (3.2-19)		15.7 (10.1-43.5)		0.02 (0-0.78)		4.44 (2.44-26.5)	
female	Pretreatment	12.3 (4.73-47.2)	<.001	31.4 (16.3-100)	<.001	0.0049 (0-0.12)	<.001	7.5 (1.08-74)	.005
	Posttreatment	5.74 (3.38-30.7)		16.5 (5.74-42.1)		0.02 (0-6.56)		4.79 (0.29-40)	
Goiter									
no goiter	Pretreatment	13.1 (4.73-44.7)	<.001	31.1 (17.3-100)	<.001	0.0049 (0-0.11)	.007	8.8 (1.96-30.2)	.015
	Posttreatment	5.56 (3.66-19.5)		16.6 (10.1-41.6)		0.01 (0-5.35)		4.51 (0.8-29.4)	
goiter	Pretreatment	12.9 (6.63-47.2)	<.001	31.1 (13.9-99.9)	<.001	0.0049 (0-0.01)	<.001	15.4 (1.82-74)	.468
	Posttreatment	5.7 (3.2-30.7)		17.5 (8.68-42.1)		0.03 (0-6.56)		7.02 (0.29-40)	
fT3 (median)									
< 12.19 pmol/L	Pretreatment	8.83 (4.73-12.2)	<.001	24 (13.9-41.9)	<.001	0 (0-0.12)	<.001	6.21 (1.82-38.7)	.022
	Posttreatment	5.77 (3.2-19)		16.9 (8.75-43.5)		0.34 (0-6.56)		4.79 (0.8-28.5)	
≥ 12.19 pmol/L	Pretreatment	22.9 (12.2-47.2)	<.001	43.3 (19.9-100)	<.001	0.0049 (0-0.12)	.001	9.7 (1.08-74)	.116
	Posttreatment	5.58 (4.05-30.7)		15.7 (5.74-42.1)		0.34 (0-6.56)		4.72 (0.29-40)	
fT4 (median)									
< 32.68 pmol/L	Pretreatment	8.92 (4.73-30.7)	<.001	23.7 (13.9-31.7)	<.001	0.0049 (0-0.12)	<.001	6.19 (1.08-38.7)	.004
	Posttreatment	5.67 (3.2-19.5)		15.9 (8.75-43.5)		0.02 (0-6.56)		4.73 (0.8-28.5)	
≥ 32.68 pmol/L	Pretreatment	22.8 (10.5-47.2)	<.001	43.3 (33.1-100)	<.001	0.0049 (0-0.05)	<.001	9.7 (2.63-74)	.265
	Posttreatment	5.6 (4.05-30.7)		17.1 (5.74-42.1)		0.03 (0-5.06)		4.85 (0.29-40)	
TRAb (median)									
< 8.48 IU/L	Pretreatment	11.3 (4.73-35.6)	<.001	28.7 (13.9-96.7)	<.001	0.0049 (0-0.12)	<.001	3.85 (1.08-8.01)	.315
	Posttreatment	5.48 (3.38-30.7)		16.5 (8.75-43.5)		0.35 (0-6.56)		3.53 (0.8-14.9)	
≥ 8.48 IU/L	Pretreatment	12.8 (5.51-47.2)	<.001	34.2 (16.3-100)	<.001	0.0049 (0-0.01)	<.001	20.5 (8.85-74)	.008
	Posttreatment	6.09 (3.2-19.5)		16.2 (5.74-41.6)		0.01 (0-5.06)		10.4 (0.29-40)	
Initial application dose of IRH (median)									
< 40 g	Pretreatment	11.05 (4.73-38.44)	<.001	28.18 (13.91-99.89)	<.001	0.0049 (0-0.12)	.003	10.69 (1.08-74.02)	.076
	Posttreatment	5.75 (3.2-30.72)		16.4 (8.79-42.05)		0.04 (0-6.56)		4.88 (0.29-37.5)	
≥ 40 g	Pretreatment	14.16 (5.34-47.17)	<.001	31.66 (17.25-100)	<.001	0.005 (0-0.11)	<.001	6.97 (1.96-38.69)	.031
	Posttreatment	5.57 (3.38-19.5)		16.23 (5.74-43.49)		0.01 (0-3.75)		4.71 (0.80-40)	
Application time of IRH (median)									
< 60 days	Pretreatment	10.18 (4.73-44.74)	<.001	28.18 (13.91-100)	<.001	0.0049 (0-0.12)	<.001	5.67 (1.08-38.69)	.380
	Posttreatment	5.77 (3.2-30.72)		17.68 (5.74-42.05)		0.02 (0-5.35)		5.03 (0.80-37.5)	
≥ 60 days	Pretreatment	14 (7.09-47.17)	<.001	33.06 (16.27-100)	<.001	0.0049 (0-0.08)	<.001	9.7 (2.08-74.02)	.004
	Posttreatment	5.58 (4.05-19.5)		15.73 (8.68-43.49)		0.06 (0-6.56)		4.76 (0.29-40)	

Additionally, due to the small sample sizes of key subgroups, *post hoc* power analyses were also conducted using G*Power 3.1 (α = 0.05, two-tailed), based on empirically observed effect sizes and actual sample sizes. *Post hoc* power exceeded 80% for fT3/fT4 in patients aged < 41 years; all parameters (fT3/fT4/TSH/TRAb) in those aged ≥ 41 years; fT3/fT4/TSH in females; fT4 in males; fT3/fT4/TRAb in non-goiter patients; and fT3/fT4 in goiter patients, indicating robust statistical power where sample sizes and effect sizes were sufficient. Moderately powered subgroups (approaching but below 80%) included: TSH in < 41years (78.0%), TRAb in females (66.0%), fT3 in males (75.8%), and TSH in non-goiter patients (76.4%). Substantial Type II error risk was observed in underpowered analyses (<60%): TRAb in < 41years (15.6%); TSH (50.3%) and TRAb (58.6%) in males; and TSH (58.0%)/TRAb (33.8%) in goiter patients, reflecting detection limitations in smaller cohorts or with reduced effect magnitudes (**Table 6**).

**Table 6:** *Post hoc* power analysis of age, sex, and goiter severity subgroups.

Subgroup	fT3		fT4		TSH		TRAb	
	Power	Cohen’s d (effect size dz)	Power	Cohen’s d (effect size dz)	Power	Cohen’s d (effect size dz)	Power	Cohen’s d (effect size dz)
Age < 41 years	99.9%	0.891	99.8%	0.852	78.0%	0.483	15.6%	0.166
Age ≥ 41 years	99.9%	1.175	99.9%	0.891	99.8%	0.852	83.6%	0.483
Women	100.00%	1.038	100.0%	1.065	99.5%	0.589	66.0%	0.306
Men	75.8%	0.89	95.9%	1.24	50.3%	0.657	58.6%	0.727
No goiter	99.9%	1.055	99.9%	1.067	76.4%	0.571	85.0%	0.639
Goiter	98.7%	0.96	98.6%	0.954	58.0%	0.486	33.8%	0.353

### 3.6 Sensitivity analysis

The sensitivity analysis was conducted to confirm the validity of our findings. First, as mentioned above, the efficacy of IRH was similar to that of ATD, as demonstrated by the changes in different serum indicators such as fT3, fT4, and TSH, as well as the regression model containing different covariates. Second, the intervention effect of IRH on thyroid function (a composite index of fT3 and fT4 analyzed together) was also equivalent to ATD (**Supplementary Table 1**). Finally, a multivariate regression analysis was employed directly on the enrolled population without PSM to explore the results of different statistical model corrections (ATD: n=167, IRH: n=73). The results exhibited that the effect of IRH on serum fT3, fT4, TSH, and TRAb levels was consistent with the previous results (**Supplementary Table 2**). In summary, the results of this study are robust.

## 4 Discussion

This retrospective study aimed to investigate the effectiveness and safety of IRH therapy, understand physicians’ preferences and habits in using IRH, and explain the preferable application conditions of IRH in treating GD patients. The patients came from the Affiliated Hospital of Liaoning University of Traditional Chinese Medicine, Shenyang, Liaoning Province, China. The hospital integrates traditional Chinese medicine culture and regional characteristics and uses traditional Chinese medicine therapies as the primary treatment strategy, which makes the study feasible. For privacy protection, the hospital data platform will not expose personally identifiable patient information. This study also passed an ethics review that waived informed consent. After screening the platform data and excluding most patients who were treated with a combination of IRH and ATD and had incomplete information, we finally obtained 240 patients who satisfied the inclusion criteria. By comparing the clinical characteristics of 167 patients in the ATD group and 73 patients in the IRH group, we noticed that the ATD group had more severe thyrotoxicosis and higher TRAb levels, intimated that doctors were more inclined to use ATD treatment when faced with severe GD, which was consistent with the consensus indications for IRH (21). The baseline characteristics of the two groups of patients in this study became homogeneous and comparable after the implementation of PSM. The overall median age was 41 years, 82% were female, and the majority had mild to moderate GD, consistent with the research group’s previous multicenter RCT and multiple studies of inorganic iodine treatment for GD (22, 23, 30, 31).

Reports of iodine monotherapy for the treatment of GD date back to the late 19th century, and numerous studies have demonstrated its effectiveness and safety in the past decade (23, 30–32). Potassium iodide (KI) can be combined with ATD to reduce the dose of ATD required in GD patients and the adverse events caused by ATD. It can also be operated alone for long-term treatment to control GD. Studies have found that iodine may be a possible alternative (33). Cases of aggravated thyrotoxicosis due to iodine intake were concentrated in areas and periods of iodine nutritional deficiency and improved with iodine nutritional supplementation (34). The prevalence of hyperthyroidism decreased because China is already an iodine-sufficient country (1, 35), in which the median urinary iodine of the residents of Shenyang was 194 μg/L (36). Studies in Asia have declared that GD patients do not benefit from decreasing their iodine intake (37, 38). With these considerations, this treatment method of great antiquity has been revitalized in China.

The recommended dose of IRH for the treatment of GD was 15 g-30 g/day of each herb (21). This study discovered that the initial dose of IRH for some patients was 10 g/day, indicating that some clinicians use a lower dose of IRH. Endocrinologists usually make an empirical judgment based on the severity of GD to apply one herb alone or two herbs of IRH in combination. Previous studies have confirmed that taking 30 g/day of Haizao and Kunbu could ensure the efficacy of IRH in a fixed-dose situation (22). Considering the scalability, this study did not restrain the dose of IRH. The dose of IRH therapy occupied in the included patients ranged from 10 g to 30 g/day, which allowed us to observe the differences in the effects of different doses of IRH in real-world treatment.

This study demonstrated that IRH could remarkably improve serum fT3, fT4, TSH, and TRAb levels, corresponding to previous studies (22). Among them, the median days required for patients from the IRH group to achieve fT3 and fT4 normalization were 33 and 41 days, respectively. As shown in **Figures 4A-C**, although there was no significant statistical difference, IRH was more rapid than ATD in ameliorating GD within 6 months. These subtle distinctions were probably due to the iodine contained in IRH. In GD, long-term iodine loading declines thyroid hormone production, iodine uptake, oxidation, and iodothyronine synthesis (39), depressing serum thyroid hormone levels preceding MMI (40, 41).

However, IRH should not be considered as a simple substitute for iodine. The amount of iodine in the pieces by Haizao and Kunbu was 814.59 μg/g and 1,182.06 μg/g, respectively (4). The dosage of IRH in this study was 10 g-30 g of each drug, and the iodine content ranged from 8.15 mg to 59.9 mg. The initial dosage of KI for the treatment of GD was 50 mg-100 mg/day, which contained 38.25 mg-76.5 mg iodine (33, 42). In addition to iodine, IRH accommodates other active ingredients such as fucosterol, isofucosterol, aurantiamide, eicosapentaenoic acid, and quercetin (15). We acquired that the proportion of serum fT4 normalization after IRH intervention was 89.0%, and the proportion of TSH normalization was about 55%, which were meliorated from the results reported in some studies on the treatment of GD with KI alone (23, 31, 43). We hypothesize that the differences in iodine content between IRH and KI, the effect of organic iodine contained in IRH is not the same as potassium iodide, and the influence of other active ingredients and the application of traditional Chinese medicine decoction on the final effect of IRH are potential causes of these differences. Further research is needed to clarify the difference between IRH and KI, which may bring new light to elucidating the mechanism of action of IRH and improving the efficacy of KI treatment.

After the effectiveness of IRH has been fully illustrated, we were also interested in the clinical characteristics of patients with poor IRH outcomes in order to identify the population to which IRH is applicable. This study described that young age, male sex, goiter, and more severe thyrotoxicosis were patient characteristics associated with unsatisfactory IRH treatment outcomes, which were well-known characteristics that adversely affect the efficacy of KI and ATD (23, 25). Nonetheless, we discovered that the IRH intervention had a noteworthy impact on the subgroup of individuals with TRAb >= 8.48 IU/L compared to the subgroup with TRAb < 8.48 IU/L. We have distinguished that practicing IRH for a long course of treatment and a higher dose can ameliorate TRAb levels.

IRH had less impaired liver function than ATD, suggesting a potentially superior safety profile. While our data capture focused on laboratory-detectable events, the multicenter RCT of 52 IRH-treated patients reported only 1 case of mild diarrhea (1.9%) and zero incidents of rash, constipation, arthralgia, or serious adverse events over 12-week follow-up (22). We assumed that IRH could be used as an alternative therapy for some GD patients with ATD intolerance.

## 5 Limitations and generalizability

The efficacy analysis of IRH should be based on the assessment of iodine’s nutritional status, as iodine is one of the active ingredients of IRH. Limited by objective circumstances, we could not assess the iodine nutrition status and thyroid iodine uptake capacity of patients, which should be improved in subsequent prospective studies. In China, potassium iodide and potassium iodate have no indications for the treatment of GD, so this study could not collect GD patients treated with inorganic iodine as one of the control groups. ATD and IRH combination or crossover dosing is common in the clinical practice of treating GD patients. Certain relevant data point could not be fully captured due to inherent constraints in the real-world retrospective data collection process, which may introduce potential confounding effects. These unmeasured factors warrant prospective validation in future well-designed studies to further strengthen the robustness of our findings. Additionally, the *post hoc* efficacy analyses demonstrated reliability in some instances. They emphasized the importance of exercising caution when interpreting the limitations of the analyses, particularly in cases involving small sample sizes or small effect sizes. We recommend that future studies aim to expand cohorts, especially in male patients. For indicators with inherently small effect sizes (e.g., TRAb), significantly larger sample sizes are necessary to ensure robust statistical testing. Nevertheless, to compare the effect of IRH therapy, this study excluded these patients. Thus, the efficacy of this treatment in the future needs to be reviewed in more depth. However, as the first retrospective cohort study of IRH for GD treatment, this study still elaborated robust results, proved the effectiveness and safety of IRH as a treatment for GD, and provided valuable experience for its subsequent promotion and use.

## 6 Conclusion

This study was operated on the intelligent research platform and effectively applied the PSM method to control for nonrandom and confounding factors in the data. IRH is an effective and safe alternative therapeutic strategy for patients with mild to moderate ATD-intolerant GD. This study summarizes the real-world experience of the clinical application of IRH and aims to lay the foundation for expanding the feasibility and popularity of IRH for treating GD.

## Data Availability

The raw data supporting the conclusions of this article will be made available by the authors, without undue reservation.
