# Supplementary material for: Therapeutic effectiveness of iodine-rich herbs in treating Graves’ hyperthyroidism: a retrospective cohort study from a single center
Source: Front Endocrinol (Lausanne). 2025 Aug 11;16:1573617. doi: 10.3389/fendo.2025.1573617 (PMC12375487; doi:10.3389/fendo.2025.1573617)
Supplement: Supplementary file 1 [file DataSheet1.docx]

**Supplementary Table 1.** Efficacy of IRH and ATD interventions in GD patients.

| **fT3+fT4** | Participants, No. | Events, No. (%) | χ2 *P* | logistic regression | | | | | | | | COX regression | |
| --- | --- | --- | --- | --- | --- | --- | --- | --- | --- | --- | --- | --- | --- |
|  |  |  |  | Crude | | model 1 | | model 2 | | model 3 | | *P* | aHR (95% CI) |
|  |  |  |  | *P* | OR (95%CI) | *P* | aOR (95%CI) | *P* | aOR (95%CI) | *P* | aOR (95%CI) |  |  |
| ATD | 51 | 73 (69.9) | 0.986 | Reference | | | | | | | | Reference | |
| IRH | 49 | 73 (67.1) |  | 0.986 | 1.007 (0.492-2.058) | 0.68 | 1.196 (0.512-2.794) | 0.996 | 0.998 (0.484-2.06) | 0.643 | 1.228 (0.515-2.929) | 0.641 | 0.99 (0.947-1.034) |

**Note:** Crude, the model only used drug intervention as the independent variable, i.e., unadjusted for confounding factors. In model 1, covariates included age, gender, and goiter; in model 2, covariates were initial fT3, initial fT4, and initial TRAb levels; in model 3, covariates were the integration of model 1 and model 2. The covariates corrected in the multivariate Cox regression model included age, gender, goiter, initial fT3, initial fT4, and initial TRAb levels.

**Supplementary Table 2.** The result of multivariate regression analysis in 167 ATD patients and 73 IRH patients.

|  | Participants, No. | Events, No. (%) | χ2 *P* | logistic regression | | | |
| --- | --- | --- | --- | --- | --- | --- | --- |
|  |  |  |  | Crude | | multivariate regression^*^ | |
|  |  |  |  | *P* | OR (95%CI) | *P* | aOR (95%CI) |
| fT3 |  |  |  |  |  |  |  |
| ATD | 140 | 167 (83.8) | 0.626 | Reference | | | |
| IRH | 63 | 73 (86.3) |  | 0.626 | 1.215 (0.555-2.661) | 0.556 | 1.382 (0.47-4.065) |
| fT4 |  |  |  |  |  |  |  |
| ATD | 139 | 167 (83.2) | 0.246 | Reference | | | |
| IRH | 65 | 73 (89.0) |  | 0.25 | 1.637 (0.707-3.788) | 0.128 | 2.739 (0.749-10.021) |
| TSH |  |  |  |  |  |  |  |
| ATD | 97 | 167 (58.1) | 0.636 | Reference | | | |
| IRH | 40 | 73 (54.8) |  | 0.636 | 0.875 (0.503-1.522) | 0.66 | 0.852 (0.418-1.736) |

**Note:** Crude, the model only used drug intervention as the independent variable, i.e., unadjusted for confounding factors. ^*^, covariates were age, gender, goiter, initial fT3, initial fT4, and initial TRAb levels.
